# Large Multinodular Toxic Goiter: Is Surgery Always Necessary?

**DOI:** 10.1155/2016/1320827

**Published:** 2016-03-23

**Authors:** Roberto Negro, Gabriele Greco

**Affiliations:** Division of Endocrinology, “V. Fazzi” Hospital, Piazza F. Muratore, 73100 Lecce, Italy

## Abstract

Patients suffering from multinodular toxic goiter (MNTG) are candidates to thyroidectomy or radioiodine 131I (131I) therapy. Thyroidectomy may be preferable especially when the volume of hyperfunctioning tissue is so large that a single administration of 131I is unlikely to cure the patient in terms of nodule's volume reduction and thyroid function. We describe the case of a 71-year-old man suffering from thyrotoxic state for the presence of two large hyperfunctioning thyroid nodules. As the patient refused surgery, at first we administered 600 MBq dose of 131I, which was unable to solve hyperthyroidism and local compressive symptoms. Then, before administering another 131I dose, the patient underwent a laser ablation treatment (LAT) in both nodules. After a significant shrinkage due to LAT, the patient received 400 MBq 131I. This procedure was able to definitely cure hyperthyroidism, to induce a significant reduction of nodules' volume, and to render the patient asymptomatic for compressive symptoms. This case demonstrates that 131I preceded by LAT represents a valid alternative strategy to surgery, even in the presence of two large coexistent hot nodules.

## 1. Introduction

Possible treatments for multinodular toxic goiter (MNTG) are radioiodine 131I (131I) therapy and thyroidectomy [1]. The decision about which of the two options is preferable relies on a number of factors like patient's preference, symptoms, nodules' volume, comorbidities, speed of recovery, side effects, and costs. In case of large hyperfunctioning nodules, thyroidectomy is generally preferred, because 131I therapy is less effective and we may need to retreat the patient [2, 3]. Surgery solves at the same time either thyrotoxicosis or compressive symptoms but needs replacement treatment with Levothyroxine and is burdened by potential complications like the risk of permanent hypoparathyroidism or recurrent laryngeal nerve injury [4]. Nonsurgical treatment like laser ablation treatment (LAT) has also been used in toxic adenoma: compared with 131I, LAT obtained a similar volume reduction but seemed inferior in normalization of serum TSH [5].

Here we present a case of 131I treatment combined with LAT for a patient suffering from a large MNTG.

## 2. Case Presentation

**Figure 1:**
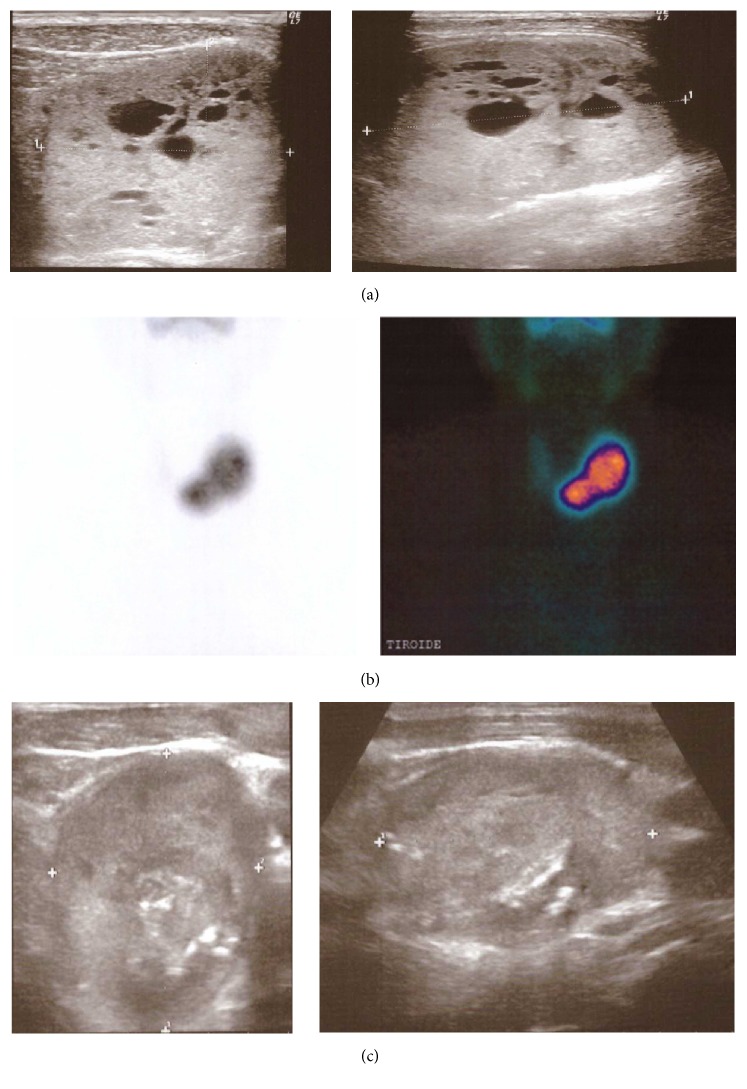
(a) Ultrasound of left lobe nodule at baseline; (b) thyroid scan (99mTc-pertechnetate); and (c) ultrasound of left lobe nodule after LAT + 131I therapy.

In September 2013 a 71-year-old man presented with thyrotoxic state. The patient suffered from hypertension and was in treatment with calcium channel blocker plus ACE inhibitor. Thyroid function test demonstrated TSH < 0.01 mIU/L, FT3: 13.7 pg/mL, and FT4: 4.1 ng/dL (normal values for TSH: 0.3–3.6 mIU/L; FT4: 0.8–1.7 ng/dL; and FT3: 2.2–4.2 pg/mL), TSH receptor antibodies and peroxidase antibodies were negative, and thyroglobulin was 337 ng/mL (normal values for TSH receptor antibodies < 4.0 U/L; peroxidase antibodies: 1–16 UI/mL; and thyroglobulin: 0.2–70 ng/mL). Thyroid ultrasound demonstrated a right lobe (volume 8 mL) with a partially cystic nodule that measured 10 × 14 × 17 mm; a spongiform nodule that measured 44 × 36 × 65 mm (volume: 55 mL) was present in the left lobe (Figure 1(a)), and another spongiform nodule that measured 31 × 25 × 34 mm (volume: 14 mL) was present in the isthmus. Thyroid scan (99mTc-pertechnetate) showed absent trapping of the right lobe, and two areas characterized by increased trapping, in the left lobe and isthmus, corresponding to the ultrasound detected nodules (hot nodules) (Figure 1(b)). A fine needle aspiration confirmed that all the three nodules were benign. Due to the remarkable volume of both isthmus left lobe nodules, the patient complained about significant compressive symptoms, especially when swallowing.

After a thorough discussion with the patient, he refused the surgical option and in November 2013 the patient underwent 131I treatment with 600 MBq on outpatient basis, although the calculated therapeutic activity exceeded by far the one administered. Thyroid function test ameliorated in the following months, but subclinical hyperthyroidism persisted (May 2014 TSH < 0.01 mIU/L and FT4: 1.6 ng/dL); the left lobe nodule decreased in volume to 40 mL and the isthmus nodule to 8.1 mL, with local compressive symptoms persistently present. In order to cure hyperthyroidism definitely, we decided to administer a further dose of 131I, but preceded by LAT to reduce the volume of the left lobe nodule. We utilised LAT in June 2014: for the left lobe nodule, we used two 75 mm, 21-gauge spinal needles and the total amount of energy delivered was 11165 Joules. As a significant liquid component was present in the nodule, it was drained just before starting LAT procedure to avoid heating of this liquid component. Six months later (December 2014) the left lobe nodule decreased to 21.5 mL and patient's function test demonstrated a persistent subclinical hyperthyroidism (TSH < 0.01 mIU/L and FT4: 1.2 ng/dL) so that 400 MBq of 131I was administered in January 2015. In February 2015 the patient showed decreased FT4 concentration (0.5 ng/dL) with normal TSH (0.42 mIU/L), and from March 2015 to date, the patient is in a condition of normal thyroid function. The left lobe nodule measures now 32 × 25.5 × 37.5 mm (volume: 17 mL) (Figure 1(c)), and the isthmus nodule measures 23.5 × 25 × 27.5 m (volume: 7.5 mL). The patient does not complain any more about compressive symptoms.

The presented case showed the efficacy of 131I treatment preceded by LAT for two coexistent large autonomous functioning nodules.

As already demonstrated, a single administration of 131I unlikely solves hyperthyroidism in a large nodule, although it may be effective in reducing its size [2, 3]. In this case, the patient had two hot nodules with an initial volume that globally was 69 mL. Indeed, one 131I dose administration was not able to cure hyperthyroidism. The use of LAT allowed significant shrinking of left lobe nodule from 40 to 21 mL, then rendering the following 131I treatment definitely effective. Recently, a prospective study demonstrated that in a single or dominant hyperfunctioning nodule, having a volume > 10 mL and at least one diameter > 30 mm, 131I preceded by LAT was more effective than 131I alone in terms of nodule's shrinkage (71 versus 47%); this strategy was also accompanied by a reduction in radioiodine-administered activity (−21%) and the resolution of hyperthyroidism was faster in the LAT + 131I group than in the 131I group [6].

In our patient, two large hot nodules were present together; a single LAT session in one of them induced a shrinkage of about 50%, then reducing the total volume of hyperfunctioning thyroid tissue and allowing 131I to be effective (at a lower dose).

In conclusion, the presented case (1) confirmed that a single administration of 131I is not effective when two large hyperfunctioning nodules are coexistent; (2) showed that the combination of LAT and 131I is a correct approach when more than 600 MBq 131I would be necessary to cure the patient; and (3) showed that the combination of LAT and 131I is rapidly effective. This strategy should be considered as a valid alternative to surgery.
